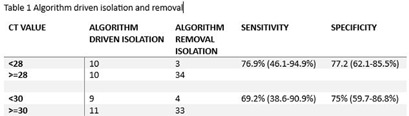# Clinical algorithm as a substitute for Ct values to predict need for isolation: can it deliver?

**DOI:** 10.1017/ash.2025.301

**Published:** 2025-09-24

**Authors:** Katherine Linsenmeyer, Judith Strymish, Rebecca Madjarov, Scott Connolly, Kristen Houghton, Kalpana Gupta

**Affiliations:** 1 VA Boston Healthcare System; 2 VA Med Ctr - West Roxbury; 3 VA Boston Healthcare System; 4 VA Boston Healthcare System; 5 VA Boston Healthcare System; 6 VA Boston and Boston University School of Medicine

## Abstract

**Background:** Many practitioners relied on SARS-CoV-2 RT-PCR cycle thresholds (Ct) to remove COVID-specific isolation given data correlating Ct values with the ability to culture live virus. Standardly, Ct values of 28-32 were used to remove isolation. However, many labs stopped reporting these values given lack of clinical validation based on a joint IDSA/AMP statement. VA Boston Healthcare System (VABHS) developed and implemented a clinical algorithm to replace Ct values to determine a need for isolation. We aimed to compare our algorithm performance to the unreported Ct results. **Methods:** We conducted a retrospective cohort study of COVID-19 PCR positive patients at VABHS between 10/1/23 and 3/31/24. During this time, VABHS required COVID-19 PCR testing (either via Cepheid Xpert Xpress CoV-2 plus or Cepheid Xpress Sars-CoV-2/flu/RSV plus) for admission regardless of symptoms. Included were all patients for whom Infectious Diseases (ID) was contacted to take off isolation using our algorithm (Fig 1). Ct values were later obtained from the lab as part of IRB-approved research to determine sensitivity of the algorithm to correctly classify isolation requirements. Ct values of 28 and 30 were used as the gold standard test for determining need for isolation. **Results:** ID was contacted to determine isolation requirements for 56 patients for whom the algorithm was applied and Ct values were later available for review. Using a Ct threshold of 28, 44 patients (78.6%) were admitted with appropriate isolation classification via the algorithm; 34 patients off isolation and 10 requiring isolation. Incorrect algorithm classification occurred for 10 patients who were isolated when not required due to lack of additional data; 2 patients who required isolation were not isolated. The algorithm failed in these 2 patients at the use of antibody results for determining time from infection; mean Ct value was 25.6 (range 23.6- 27.6). Both patients had COVID within the last 30 days and positive antibody testing. The true positive rate/sensitivity for algorithm driven isolation was 83.3% (51.6-97.9%) and the true negative rate/specificity for algorithm driven removal of isolation was 77.3% (62.1-88.5%). When the Ct threshold is modified to 30, the sensitivity and specificity of the algorithm were 78.6% (49.2-95.3%) and 78.6% (63.2-89.7%) (table 1). No transmissions occurred using the algorithm during this study period. **Conclusions:** Strategic use of an algorithm using history, antigen and antibody results was moderately accurate compared to Ct values for assessing isolation requirements. No known transmissions occurred with use of the algorithm in lieu of Ct values.

**Figure:**
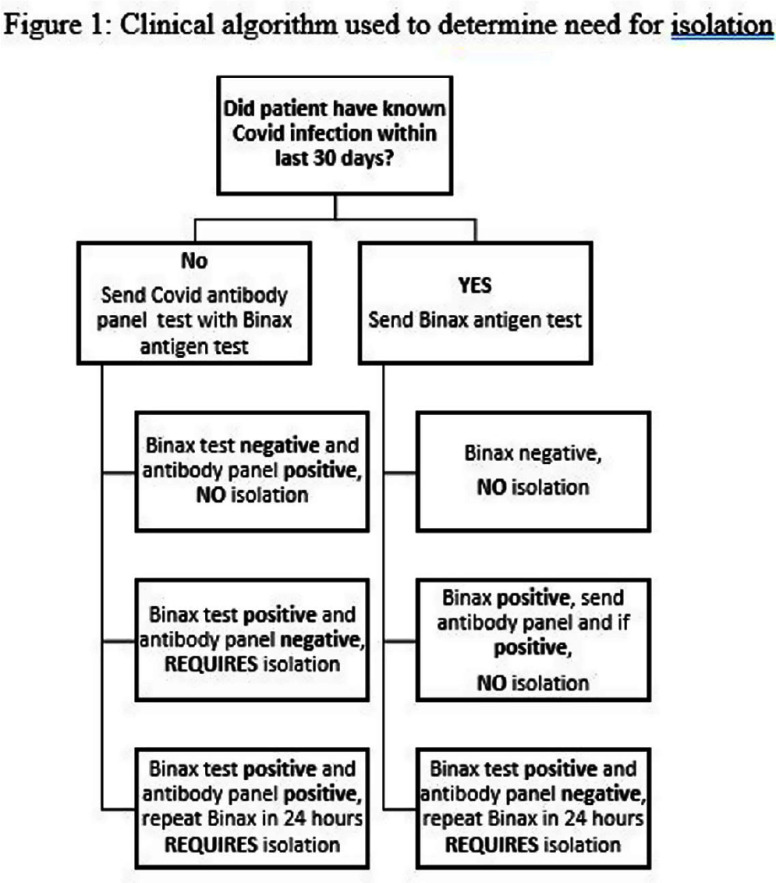

**Figure:**